# Genome wide-analysis of anterior-posterior mRNA localization in *Stentor coeruleus* reveals a role for the microtubule cytoskeleton

**DOI:** 10.1101/2023.01.09.523364

**Published:** 2023-01-10

**Authors:** Ashley R. Albright, David Angeles-Albores, Wallace Marshall

**Affiliations:** 1 Department of Biochemistry and Biophysics, University of California, San Francisco, San Francisco, CA, USA; 2 Center for Cellular Construction, University of California, San Francisco, San Francisco, CA, USA; 3 Altos Labs, San Francisco, CA, USA; 4 Twitter: @WallaceUCSF

## Abstract

Cells have complex and beautiful structures that are important for their function, but understanding the molecular mechanisms that produce these structures is a challenging problem due to the gap in size scales between molecular interactions and cellular structures. The giant ciliate *Stentor coeruleus* is a unicellular model organism whose large size, reproducible structure, and ability to heal wounds and regenerate has historically allowed the formation of structure in a single cell to be addressed using methods of experimental embryology. Such studies have shown that specific cellular structures, such as the oral apparatus, always form in specific regions of the cell, which raises the question of what is the source of positional information within this organism? By analogy with embryonic development, in which localized mRNA is often used to mark position, we asked whether position along the anterior-posterior axis of Stentor might be marked by specific regionalized mRNAs. By physically bisecting cells and conducting half-cell RNA-sequencing, we were able to identify sets of messages enriched in either the anterior or posterior half. We repeated this analysis in cells in which a set of longitudinal microtubule bundles running down the whole length of the cell, known as KM-fibers, were disrupted by RNAi of β-tubulin. We found that many messages either lost their regionalized distribution, or else switched to an opposite distribution, such that anterior-enriched messages in control became posterior-enriched in the RNAi cells, or vice versa. This study indicates that mRNA can be regionalized within a single giant cell, and that microtubules may play a role, possibly by serving as tracks for movement of the messages.

## Introduction

Understanding how complex patterns arise within individual cells is a challenging biological problem due to the vast difference in spatial scales from molecules to whole cells [1,2]. The questions that arise in understanding cellular morphogenesis are essentially the same as those that arise in understanding development of embryos - how does the system know which structures to make and how to position them relative to an overall body plan. A classic model organism that facilitates the study of developmental biology in a single cell is the giant ciliate *Stentor coeruleus* [3]. *Stentor* cells are 1 millimeter long and covered with blue stripes of pigment, which alternate with longitudinal rows of cilia consisting of basal bodies associated with microtubule bundles (KM-fibers) that run the length of the cell. The cone-shaped cell has an array of cilia known as the oral apparatus (OA) at its anterior end, and a holdfast at the posterior. Orthogonal to the anterior-posterior (AP) axis defined by these structures, the pigment stripes show a graded distribution of width, defining a circumferential axis. Starting with the widest stripes, the stripe width gradually decreases as one moves counter-clockwise around the circumference, until eventually the narrowest stripes meet the widest stripes, creating a discontinuity known as the contrast zone.

Part of the reason that *Stentor* was first used as a model system is its ability to regenerate after surgical manipulations. If any part of the *Stentor* cell is cut off, the missing piece regenerates in a matter of hours to yield a normal cell. If a cell is cut in half, each half regenerates normal structure [4,5]. Part of the reason that *Stentor* can regenerate from these surgical operations is that it contains an elongated macronucleus that contains many copies of every gene. Another reason Stentor is able to regenerate is its ability to employ a range of mechanical wound-closure mechanisms to keep its cytoplasm from leaking out while it patches over a wound [6]. *Stentor’s* huge size, easily visible patterning, and amazing powers of regeneration, attracted many developmental biologists during the turn of the last century, including Thomas Hunt Morgan [5]. For almost a century, up until the 1970’s, microsurgeries were used to analyze morphogenetic processes in *Stentor*, resulting in a wealth of information about how cells responds to geometrical re-arrangements [7,8]. But *Stentor* was never developed as a molecular model system, and we do not currently know the molecular basis for how it is able to regenerate its structures. In order to exploit the unique opportunities presented by this organism, we sequenced the *Stentor* genome [9], developed methods for RNAi by feeding [10], and analyzed the transcriptional program of regeneration [11].

Perhaps the most fundamental question about pattern formation in any organism is what is the source of positional information. That such information exists in *Stentor* can be inferred from the fact that cellular structures always form in defined and reproducible positions [7]. For example, during regeneration or normal cell division, the new OA always forms halfway down the AP axis at the contrast zone between narrow and wide stripes [12]. It is generally true in ciliates that specific surface structures form at positions that can be defined with respect to a cylindrical coordinate system [13–15]. In this system, position is defined by two orthogonal axes - an AP axis that runs along the length of the cell, and a circumferential axis that runs around the equator, which in Stentor is manifest by the gradient of pigment stripe width. We have previously analyzed the distribution of the proteome relative to the AP axis, by cutting individual Stentor cells into pieces and analyzing the fragments by mass spectrometry [16]. This study found that approximately 25% of the proteome was polarized relative to this axis.

The role of localized mRNA as a source of positional information in embryonic development is well established, particularly in *Drosophila*. We anticipate that regionalized RNAs in different parts of the *Stentor* cell will be important sources of positional information, just like they are in embryos. Unlike the case of *Drosophila* development, where a number of important genes had already been identified in genetic experiments, we do not currently have a list of candidate positional information molecules to map. Inspired by prior work in cryoslicing *Drosophila* embryos to obtain positional information [17,18], we can use a similar unbiased approach to map the entire transcriptome to identify genes that might have a regionalized distribution.

Modern studies address sub-cellular localization of transcripts with single-molecule (smFISH [19]) or multiplexed error-robust fluorescence *in situ* hybridization (merFISH [20]). Many studies that survey message localization by FISH do so in cells that lack an obvious polarity axis, so collecting physical fragments of cells representing specific regions and mapping fragments back to any reference frame would not be possible. Consequently, in those cases, each message must be visualized sequentially by imaging, which is time-consuming and expensive. The giant size of *Stentor*, combined with the reproducible cell geometry and easily visualized body axis markers, makes it possible to use a sequencing-based method to map RNA localization relative to the body axis coordinates. The first question is whether genes are, in fact regionalized in *Stentor*. If it turns out that RNAs are localized to different parts of the *Stentor* cell, the next question is how might this be achieved?

**Figure 1 –:**
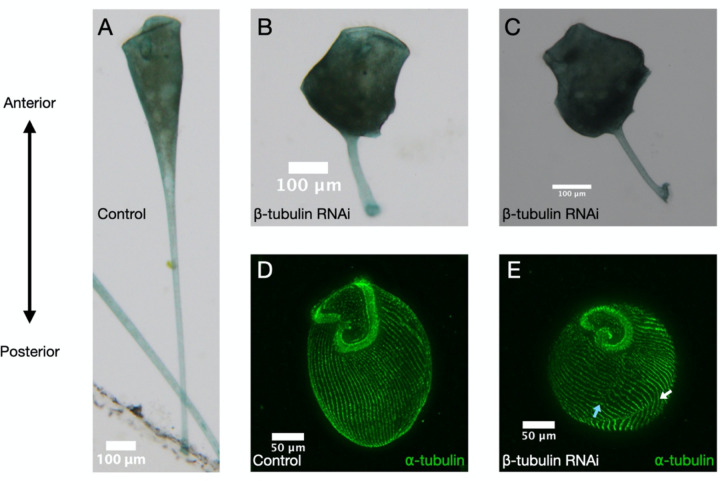
β-tubulin knockdown results in loss of typical cell morphology and orderly microtubule bundles Brightfield images of (A) control and (B, C) β-tubulin knockdown cells showing disruption of typical cell morphology. Immunofluorescence of α-tubulin stained (green) in (D) control and (E) knockdown cells show that cells have broken (blue arrow) and discontinuous (white arrow) rows of microtubule bundles. Cells are oriented with the anterior at the top and posterior at the bottom. All images in this figure are adapted from Slabodnick et al 2014 [10].

The surface of a *Stentor* is covered with parallel microtubule bundles with the plus end at the anterior and minus end at the posterior. Studies in other systems from yeast to human neurons show that the cytoskeleton is involved in active RNA transport[21,22], therefore we hypothesize that transcripts in this giant cell may be polarized by trafficking specific messages along the cell’s polarized cytoskeletal tracks. RNAi knockdown of β-tubulin, a major component of the microtubule cytoskeleton, causes *Stentor* to lose their characteristic trumpet shape (Fig 1A-C [10]) and lose their orderly microtubule bundles (Fig 1D-E [10]), but such cells are still alive and retain an OA at one end and a holdfast at the other. It is thus still possible to recognize the AP axis in such cells, and ask whether disruption of the microtubule tracks hypothesized to carry messages that has any effect on localization.

Here, we investigate both the disruption of messages and the microtubule cytoskeleton in transcript polarity by conducting half-cell RNA sequencing in paired anterior and posterior halves of *Stentor coeruleus* following lateral bisection. Overall, we find evidence for a global disruption of transcript polarity as well as identify 346 candidate transcripts with an anti-correlated shift in AP skew.

## Methods

### Stentor culture, RNAi, and bisection

*Stentor coeruleus* were obtained from Carolina Biological Supply and maintained in small Pyrex dishes at room temperature in filter-sterilized pasteurized spring water (Carolina Biological Supply – Burlington, NC) subsequently referred to as Carolina Spring Water (CSW). Twice a week, a lentil-sized pellet of *Chlamydomonas reinhardtii* grown on TAP plates were resuspended in CSW and fed to *Stentor*. Prior to RNAi, *Stentor* were isolated from the main culture in 6-well tissue culture plates containing 2 mL CSW, starved for 3 days, and washed 3 times with CSW eliminate residual *Chlamydomonas.*

β-tubulin RNAi was conducted by feeding as previously described[10] with minor changes. The sequenced targeted was amplified with the following oligos: forward - 5’ ATGGTGACTTAAATCACTTGGTTAGTGC 3’, reverse – 5’ TTAAGCAGCTTCCTCTTCCTCATCG 3’. Transformed HT115 *E. coli* were grown to log phase and expression of dsRNA induced with 1 mM IPTG overnight at room temperature. Overnight 50 mL cultures were spun down, washed with CSW, and resuspended in 30 mL of CSW. Aliquots of 1 mL were pelleted, with as much supernatant as possible removed, and frozen at −80° for future use. For feeding, pellets were resuspended in 1 mL CSW. *Stentor* were fed 500 uL control (empty vector) or β-tubulin RNAi bacteria once a day for 7 days.

Individual *Stentor* were washed and transferred to a 2 uL droplet of 1.5% methylcellulose in CSW and bisected laterally using a glass needle. Halves were pipetted up and down in CSW to remove residual methylcellulose prior to processing.

### Half-cell RNA-sequencing

Within 20 minutes of bisection, cells were lysed and prepped for sequencing with the NEBNext Single Cell/Low Input RNA Library Prep kit for Illumina (Cat. No. E6420) according to the manufacturer’s protocol. Samples were pooled and submitted for sequencing. Sequencing on an Illumina NovaSeq6000 SP200 was performed at the UCSF CAT, supported by UCSF PBBR, RRP IMIA, and NIH 1S10OD028511–01 grants.

### Data processing and analysis

Raw data will be available on Dryad following approval here: https://doi.org/10.6078/D1WT6W

Data were processed and analyzed with custom scripts in both Python and R, which are available here: https://github.com/aralbright/2022_AADAWM

Adapter trimming was performed using flexbar [23] as instructed by NEB for the NEBNext Single Cell Library Prep Kit: https://github.com/nebiolabs/nebnext-single-cell-rna-seq. We then used kallisto[24] to generate a reference index and quantify transcript abundance. Using sleuth [25], we obtained normalized transcripts per million (TPM). TPM values were log(1+x) transformed. We performed a principal components analysis (PCA) to visualize relatedness of the samples. To reduce effects of outlying transcript abundance on significance, we regularized mean log-transformed TPM values (subsequently referred to as log1 Regularized Transcripts Per Million, or log1RPM). Anterior-posterior skew is then defined as the difference over the sum of log1RPM.


$$Skew\;=\frac{A_{log1RPM}-P_{log1RPM}}{A_{log1RPM}+P_{log1RPM}}$$

Genes were selected for further analysis that passed a minimal cutoff of abundance, to ensure sufficiently robust statistical comparisons, and it was also required that genes had a coefficient of variation (variance divided by the mean) less than 3, in order to remove genes with such variable distributions that comparisons might be unreliable. For genes meeting these criteria, skews were compared for both control and β-tubulin RNAi samples.

We were particularly interested in genes having anti-correlated skews, which are the most interesting candidates for disrupted localization. Such genes were selected with feature selection by PCA, in which the genes were treated as factors and the samples data points, to identify linear combinations of genes that give the highest variation among the samples. Feature selection was then followed by a t-test for significance.

## Results

In order to ask whether any genes have localized distribution relative to the AP axis, we manually cut *Stentor* cells in half, using the OA as a marker to define the anterior end, and cutting approximately mid-way between the OA and the other end of the cell. For each dissected cell, the anterior half and posterior half cells were used to build libraries for RNA sequencing. We then define skew in terms of the difference in abundance of transcripts between the two half-cells in each sample. To quantify polarization along the AP axis, we log(1+x) transformed and regularized normalized transcripts per million. Then, we calculated the difference over the sum of AP values for each gene within each sample to obtain a skew value(see Methods for details). We removed genes with highly variable expression between samples or expression levels at the extreme end of high or low in order to ensure that these characteristics are not affecting our overall calculation of skew. We expect that skews will be variable to some extent even in normally polarized cells, given that the precision of bisection is subject to human error. With this analysis, each gene is assigned an average skew, taken over all samples, with positive values reflecting anterior enrichment and negative values reflecting posterior enrichment.

Among the 100 genes showing the largest absolute skew, 80% had negative skew, suggesting that there may be a higher degree of regionalization in the posterior half of the cell. Among the genes showing a strong negative skew are SteCoe_19554, encoding an ortholog of DisA/SF-Assemblin, SteCoe_9217 encoding a cytoplasmic dynein light chain, and SteCoe_14908, encoding a hypothetical protein corresponding to a gene upregulated during regeneration of the OA. Most of the skewed genes encode hypothetical proteins. Understanding what functions these uncharacterized proteins may play in Stentor pattern formation is an interesting future goal, but here our primary interest is in the mechanisms by which the localization is achieved.

**Figure 2 –:**
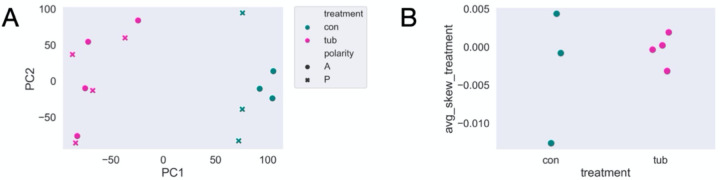
Polarity disruption suggested by PCA and average skew in control vs. β-tubulin knockdown samples (A) PCA of normalized transcripts per million (TPM) across entire samples. Control labeled in green and β-tubulin knockdown in magenta. Anteriors are represented by circles and posteriors by ‘x.’ (B) Average skew of all genes within each sample.

Given that the surface of Stentor is covered with parallel, oriented microtubule bundles, an obvious hypothesis is that motors might move transcripts to one end or the other of these bundles. To understand the role of the microtubule cytoskeleton in cellular patterning, we conducted half-cell RNA-sequencing of paired anteriors and posteriors in control and β-tubulin knockdown *Stentor coeruleus*. As expected, PCA shows a clear separation between control and β-tubulin samples on the first principal component (Fig 2A). Interestingly, anterior and posterior (AP) control samples are separated by the first component while AP β-tubulin samples are not. These differences at the whole-sample suggest that the microtubule cytoskeleton is broadly important for proper RNA localization, such that anterior and posterior samples become more similar to each other in the absence of the microtubule tracks.

We observed that the mean, the mean skew of all genes is more variable across control than β-tubulin samples (Fig 2B). This might be expected if there was a general loss of polarized localization in the β-tubulin RNAi, such that variation in the exact position of cutting the cell would have less of an effect on the average skew.

**Figure 3 –:**
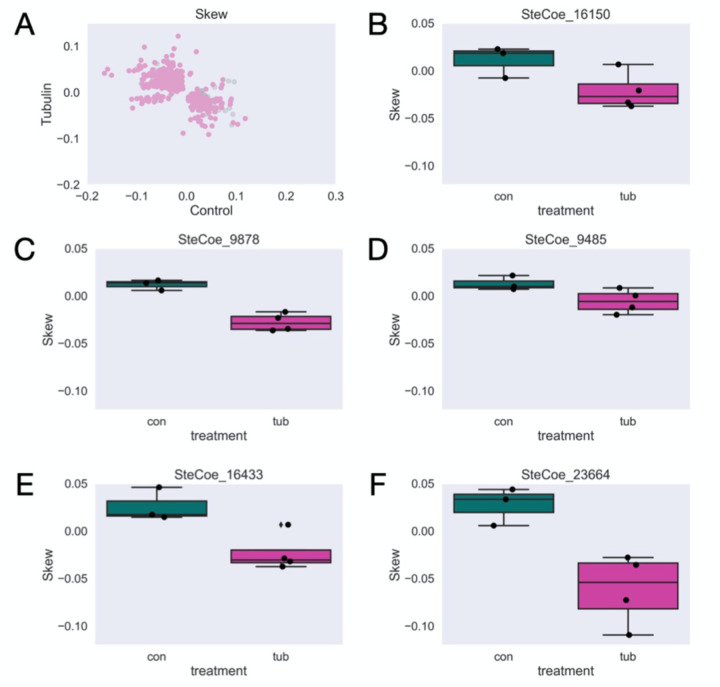
Significant anti-correlated skews between anterior and posterior halves yields 346 candidates for transcripts with disrupted localization (A) Average skew for each gene across all control versus β-tubulin samples after filtering. Significantly skewed candidates are colored in pink. (B-E) Representative examples of genes with significantly different skews between control and β-tubulin knockdown cells.

If microtubule tracks worked to separate a subset of transcripts along the AP axis, one would expect loss of skew in the RNAi cells, which is certainly seen for many genes. For other genes, in which the skews remain correlated, it suggests that some degree of regionalization is still possible without intact microtubule tracks. The track model can thus account for loss of correlation or retention of positive correlation. A more surprising outcome would be anti-correlation, such that a gene switches from anterior enriched in the control to posterior enriched in the knockdown, or vice versa. To look for such genes, we performed feature selection by PCA to detect genes with anti-correlated skews (see Methods). Although changes in skew that remain correlated may be significant (i.e. cases where an anterior-expressed gene still has an anterior skew, but perhaps more extreme), candidates with anti-correlated skews upon β-tubulin knockdown are the most interesting for further analysis as these account for the largest differences in skew between control and β-tubulin cells. Following feature selection, we found 346 candidates out of 500 tested with a significant anti-correlated skew (Fig 3A, Supplementary Table 1). Five of these candidates were previously found to be enriched in the anterior or posterior in a previous proteomic study [16]. We show these here as representative examples of candidates found in our study (Fig 3B-F). Interestingly, each of these candidates has a negative, or more posterior, shift in skew upon β-tubulin knockdown despite 4 out of the 5 corresponding proteins are more enriched in the posterior half of the cell under normal conditions. Other potential candidates for further study include the most positive (Table 1) and most negative (Table 2) differences in skew between control and β-tubulin RNAi.

## Discussion

Here, we provide evidence that the microtubule cytoskeleton is broadly important for proper RNA localization in the giant ciliate, *Stentor coeruleus.* Knockdown of β-tubulin, a major component of the microtubule cytoskeleton, disrupts normal cell morphology and causes cells to lose their orderly microtubule bundles (Fig 1, [10]). Half-cell RNA-sequencing in paired anteriors and posteriors of the cell also reveals global disruptions in polarity by PCA and in a more variable skew present in control versus β-tubulin knockdown cells (Fig 2). Overall, we find many candidates with significant and anti-correlated skews (Fig 3). Five of these candidates were found to be enriched in one half over the other in a previous proteomics study (Fig 3B-F, Lin et al 2022[16]). We speculate that these candidates may be important for cellular patterning given the different enrichment as well as significantly different skew in expression upon β-tubulin knockdown where cell polarity is disrupted.

This work shows that transcript patterning is disrupted upon the loss of β-tubulin and disruption of the microtubule cytoskeleton, but raises several additional questions: (1) Are the candidates we found directly involved in cellular patterning or the regeneration of new patterned structures? (2) Is the microtubule cytoskeleton essential for regeneration given its role in patterning the cell? (3) Does protein enrichment in one half over the other arise from differential transcript localization or possibly translation? As we work on developing additional tools to validate this study, such as smFISH, we plan to conduct RNAi of the 5 representative candidates mentioned to assay for morphological and/or regeneration defects. Other candidates include for study include those listed in Table 1, Table 2, and all 346 in Supplementary Table 1. Work to improve the genome and transcriptome is also ongoing, one limitation of this study is the percent pseudoalignment is low ranging from 10–20%, or 4–9 million reads per sample. We do expect lower than typical pseudoalignment as ribosomal DNA is present at over 1 million ploidy in *Stentor* [9]; however, we expect that we may still find additional candidates from this experiment in the future with an improved genome and transcriptome. Regardless, we are encouraged by the results we found despite a relatively low number of reads per sample.

**Figure 4.**
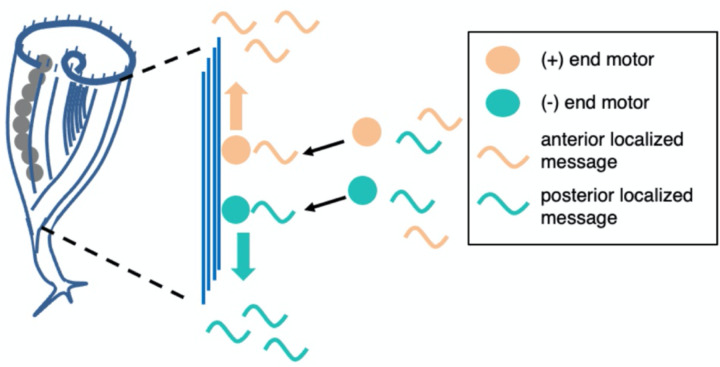
Model for RNA localization in *Stentor* via motor-directed transport.

RNA localization could be a key to understanding pattern formation and regeneration in *Stentor*. Our results suggest a potential model for how this localization is achieved. Given that disruption of the cortical microtubule bundles led to a loss or reversal of polarized localization for many messages, we propose that messages associate with microtubule motors via RNA-binding adaptors, such as BicD, and then move to the plus or minus ends of the bundles (Figure 4). Future work will be needed to test this model by interfering with motors and potential adaptor proteins.

As this is a preprint-in-progress, we welcome input on additional interesting candidates for further analysis, as well as any feedback or other suggestions for this manuscript.

## Supplementary Material

Supplement 1

## Figures and Tables

**Table 1.** Top 10 positive-shifted (more anterior) skews

Gene	Skew	Protein Domain
SteCoe_37198	0.103	Cystatin domain protein
SteCoe_23639	0.069	WD40 domain protein
SteCoe_3835	0.067	SBP_bac_3 domain protein
SteCoe_31769	0.067	kinase related to Ciliate-E2-Unclassified subfamily
SteCoe_164	0.062	AMPK subfamily kinase
SteCoe_2990	0.060	STE related kinase
SteCoe_22530	0.057	TPR_2 domain protein
SteCoe_1329	0.057	RIH_assoc domain protein
SteCoe_4636	0.056	MAT1 domain protein
SteCoe_14610	0.055	CK_II_beta domain protein

**Table 2.** Top 10 negative-shifted (more posterior) skews

Gene	Skew	Protein Domain
SteCoe_26054	−0.116	zf-LITAF-like domain protein
SteCoe_27980	−0.095	CLN3 domain protein
SteCoe_10036	−0.092	CK1 kinase
SteCoe_17481	−0.089	CDPK-like predicted pseudokinase
SteCoe_14494	−0.089	zf-RING_2 domain protein
SteCoe_22865	−0.087	TCR domain protein
SteCoe_6843	−0.086	ABC_tran domain protein
SteCoe_22502	−0.086	PX domain protein
SteCoe_7554	−0.075	Thioredoxin_8 domain protein
SteCoe_8635	−0.074	MFS_1 domain protein
